# Associations of Polymorphisms in DNA Repair Genes and MDR1 Gene with Chemotherapy Response and Survival of Non-Small Cell Lung Cancer

**DOI:** 10.1371/journal.pone.0099843

**Published:** 2014-06-16

**Authors:** Yan Du, Tong Su, Lijun Zhao, Xiaojie Tan, Wenjun Chang, Hongwei Zhang, Guangwen Cao

**Affiliations:** 1 Department of Epidemiology, Second Military Medical University, Shanghai, China; 2 Department of Pulmonary Medicine, Changhai Hospital, Second Military Medical University, Shanghai, China; MOE Key Laboratory of Environment and Health, School of Public Health, Tongji Medical College, Huazhong University of Science and Technology, China

## Abstract

**Objectives:**

We aimed to determine the associations of genetic polymorphisms of excision repair cross-complementation group 1 (*ERCC1*) rs11615, xeroderma pigmentosum group D (*XPD*/*ERCC2)* rs13181, X-ray repair cross complementing group 1 (*XRCC1*) rs25487, *XRCC3* rs1799794, and breast cancer susceptibility gene 1 (*BRCA1*) rs1799966 from the DNA repair pathway and multiple drug resistance 1 (*MDR1/ABCB1*) rs1045642 with response to chemotherapy and survival of non-small cell lung cancer (NSCLC) in a Chinese population.

**Materials and Methods:**

A total of 352 NSCLC patients were enrolled to evaluate the associations of the six SNPs with response to chemotherapy and overall survival. Logistic regressions were applied to test the associations of genetic polymorphisms with response to chemotherapy in 161 advanced NSCLC patients. Overall survival was analyzed in 161 advanced and 156 early stage NSCLC patients using the Kaplan-Meier method with log-rank test, respectively. Multivariate Cox proportional hazards model was performed to determine the factors independently associated with NSCLC prognosis.

**Results:**

*BRCA1* rs1799966 minor allele C (TC+CC *vs.* TT, OR = 0.402, 95%CI = 0.204−0.794, p = 0.008) and *MDR1/ABCB1* rs1045642 minor allele A (GA +AA *vs.* GG, OR = 0.478, 95%CI = 0.244−0.934, p = 0.030) were associated with a better response to chemotherapy in advanced NSCLC patients. Survival analyses indicated that *BRCA1* rs1799966 TC+CC genotypes were associated with a decreased risk of death (HR = 0.617, 95% CI = 0.402−0.948, p = 0.028) in advanced NSCLC patients, and the association was still significant after the adjustment for covariates. Multivariate Cox regression analysis showed that *ERCC1* rs11615 AA genotype (*P* = 0.020) and smoking (p = 0.037) were associated with increased risks of death in early stage NSCLC patients after surgery.

**Conclusions:**

Polymorphisms of genes in DNA repair pathway and *MDR1* could contribute to chemotherapy response and survival of patients with NSCLC.

## Introduction

Lung cancer is one of the leading causes of cancer deaths worldwide [1]. Non-small-cell lung cancer (NSCLC) is the most common subtype and accounts for 85% of all lung cancer. Most NSCLC patients are at developed advanced tumor stage upon diagnosis and lose the opportunity of surgical resection [2]. Platinum-based combined chemotherapy is a standard treatment for advanced NSCLC. However, the outcome and survival of advanced NSCLC are generally poor, with a 5-year survival rate of only about 15%. In addition, the survival rate of advanced NSCLC varies greatly in different populations with diverse genetic background [3], [4]. Furthermore, the therapeutic efficacy of platinum-based regimens was affected by drug resistance. Possible mechanisms of chemoresistance include alterations in drug efflux or influx, DNA repair capacity, and other cellular pathways required for response to DNA damage. For patients with early stage NSCLC that can be surgically excised, the long-term prognosis is not satisfactory and the 5-year survival rate after surgery is less than 50% [5]. The outcome of early stage NSCLC patients receiving surgical treatment is also closely related to individual genetic characteristics, since variation and expression levels of certain genes can affect the survival and adjuvant chemotherapy response [6], [7]. Genetic variation analysis in candidate pathways has shown that an individual's genetic background plays an important role in disease development, treatment response, and survival. For example, patients with completely resected NSCLC and negative expression of excision repair cross-complementation group 1 (ERCC1) protein in tumors benefit more from adjuvant cisplatin-based chemotherapy than those with ERCC1 positive expression [8]. Previous studies have shown that tumor-node-metastasis (TNM) staging system, age, performance status and weight loss are associated with NSCLC prognosis; however, the predictive powers of these factors are not optimal. Therefore, it is crucial to identify new biomarkers that can improve prognostic and predictive assessment accuracy to help developing personalized cancer treatment and patient-tailored chemotherapy, and eventually achieving better outcomes for NSCLC patients.

DNA repair capacity (DRC) is a double-edged sword in cancer etiology and treatment. Defects in DNA repair system drastically increase cancer risk [9]. On the other hand, DNA repair mechanism may reduce the therapeutic efficacy of chemotherapy by allowing cancer cells to fix DNA damages caused by these agents [10]. DNA repair involves coordination of many genes in four major DNA repair pathways, which are nucleotide excision repair (NER), base excision repair (BER), double-strand break repair (DSBR), and mismatch repair (MMR) pathways. Single nucleotide polymorphisms (SNPs) in DNA repair genes may modulate DNA repair capacity via influencing protein expression or activities, and therefore affecting lung cancer risk [11]–[13]. DNA repair gene polymorphisms may be associated with the risk and prognosis of NSCLC. Xeroderma pigmentosum group D (*XPD*/*ERCC2)* polymorphism can alter the secondary structure of mRNA [14]; and SNP in the X-ray repair cross complementing group 1 (*XRCC1*) gene 5′ untranslated region (UTR) greatly enhance trans-activator Sp1 binding element, therefore decreasing promoter activity and decreasing protein expression [15]. Furthermore, polymorphisms in these genes may also be potential prognostic markers for both chemotherapy response and overall survival of NSCLC patients [16]–[19].

Multiple drug resistance 1 (*MDR1*, also known as *ABCB1*) gene encodes P-glycoprotein (P-gp), a transmembrane transporter belonging to the ATP-binding cassette family, which can pump out intracellular chemotherapeutics and induce multiple drug resistance. *MDR1/ABCB1* C3435T (rs1045642) polymorphism is associated with expression level and function of MDR1 [20], thus may affect the responses to anticancer drug treatment.

In this study, we selected six previously studied SNPs, located either on the protein coding regions or in regulatory regions of the genes, including *ERCC1* rs11615, *XPD/ERCC2* rs13181 and breast cancer susceptibility gene 1 (*BRCA1*) rs1799966 from the NER pathway; *XRCC1* rs25487 from the BER pathway, *XRCC3* rs1799794 from the DSBR pathway, as well as *MDR1/ABCB1* rs1045642 (Table S1). We first investigated the associations between these SNPs and chemotherapy response in advanced NSCLC patients, then evaluated their relationships with the survival of NSCLC patients who received surgical treatment or chemotherapy.

## Materials and Methods

### Study population

The participants were recruited at Changhai Hospital of the Second Military Medical University. A total of 352 primary NSCLC patients diagnosed from July 1997 to October 2008 were enrolled (Dataset S1). All patients were histopathologically confirmed as NSCLC. Clinical and histopathologic data of patients were extracted from their medical records. All patients were of ethnic Han Chinese origin. The study protocol conformed to the ethical guidelines of the Declaration of Helsinki (2000), and was approved by the Institutional Review Board of this university. All participants provided written informed consent.

### Data collection and follow-up

Of the 352 NSCLC patients, 161 (45.7%) advanced NSCLC patients received evaluable platinum-based chemotherapy. Besides cisplatin (DDP) or carboplatin (CBP), chemotherapy regimens also included gemcitabine (GEM), paclitaxel (TAX), docetaxel (DOC), vinorelbine (NVB), or pemetrexed disodium (PEM). Drug dosages were: DDP 75 mg/m^2^ on day 1; CBP area under curve (AUC)  = 5−6 g on day 1; GEM 1250 mg/m^2^ on days 1 and 8; TAX 135∼175 mg/m^2^ on day 1 over 3 hours; DOC 75 mg/m^2^ on day 1 over 1 hour; NVB 25 mg/m^2^ on days 1 and 8; PEM 500 mg/m^2^ on day 1. All drugs were administrated by intravenous infusion every 3–4 weeks for a treatment cycle. Tumor response was assessed after 2 treatment cycles according to the Response Evaluation Criteria in Solid Tumors [21]. Responses were classified into complete response (CR), partial response (PR), stable disease (SD) or progressive disease (PD). Patients with CR, PR or SD were defined as “patients with clinical benefit”, and patients with PD were defined as “patients without clinical benefit” [22].

Follow-up was started 2 months after the definite diagnosis. Follow-up was performed by telephone or in-person interview at the outpatient department at an interval of 3 months according to our standard epidemiological procedure. Median follow-up period was 20.8 months (range: 1.0 to 178.6 months).

### Genotyping

Genomic DNA was extracted from peripheral blood using QIAmp DNA extraction kits (QIAGEN, Hilden, Germany). SNPs were genotyped using fluorescent-probe real-time quantitative PCR (qPCR) in a LightCycler^TM^480 (Roche, Basel, Switzerland). Primers and probes (Taqman or Minor Groove Binder [MGB]) were designed and synthesized by GeneCore BioTechnologies (Shanghai, China). The sequences of primers and probes are available in Table S1. Each reaction mixture contained 0.2 µmol/L of primers, 0.2 µmol/L of probes, 0.1 µg–0.5 µg purified templates in Premix Ex Taq reaction system (Takara, Dalian, China). The reactions were programmed at 95°C for 10 s and followed by 40 cycles of 95°C for 10 s and 60°C for 30 s. All samples were successfully genotyped (Dataset S1). For quality control, 5% samples were randomly selected and directly sequenced, and 100% identical results were obtained.

### Statistical analysis

Logistic regression analysis was performed to obtain odds ratios (ORs) and their 95% confidence intervals (95% CIs) for the relationships of genetic polymorphisms with chemotherapy response in 161 advanced NSCLC patients. Overall survival (OS) was analyzed using the Kaplan-Meier method in 161 advanced and 156 early stage NSCLC patients separately. Log-rank test was used to compare the survival curves. Forward stepwise multivariate Cox proportional hazards model (*P*_entry_ = 0.05, *P*_removal_ = 0.10) was performed to determine the factors contributing independently to NSCLC prognosis and estimate the hazard ratios (HRs) and their 95% CIs. Further subgroup analyses stratifying by histology, smoking status, Eastern Cooperative Oncology Group (ECOG) performance status, and stage were also performed. Covariates were adjusted to obtain HRs and corresponding 95% CIs in subgroup analyses. All statistical tests were two-sided and conducted using Statistical Program for Social Sciences (SPSS 16.0 for Windows, SPSS, Chicago, IL). *P*<0.05 was considered as statistically significant.

## Results

### Patient characteristics

The demographic and clinical characteristics of the patients are shown in Table 1. Of the 352 NSCLC cases, 156 (44.3%) were early stage patients who underwent surgical treatment, 161 (45.7%) were advanced patients at an inoperable stage and received platinum-based chemotherapy, 10 (2.8%) patients refused any treatment for personal reasons, and 25 (7.1%) patients lost to follow up after the first treatment at Changhai Hospital. Most (120/156, 76.9%) early stage NSCLC patients who underwent surgery received preoperative or postoperative adjuvant chemotherapy. Advanced NSCLC patients were more likely to receive radiation therapy compared to early stage patients (44.1% *vs.* 20.5%, p<0.0001). Other demographic and clinical characteristics were similar between early stage and advanced NSCLC patients (p>0.05 for all). Table S2 presented the genotype distributions of these six SNPs in different categories of the NSCLC patients.

**Table 1:** Demographic and clinical characteristics of the NSCLC patients.

	Total (n = 352)	Classified according to treatment and follow-up results				
		Early stage patients with surgery (n = 156)	Advanced patients with chemotherapy (n = 161)	Patients without treatment (n = 10)	Patients lost to follow up (n = 25)	*P* *
Age (year)						0.78
Range	26–90	26–84	30–90	56–76	35–78	
Mean ± SD	59.5±10.6	59.2±9.9	58.9±11.3	66.7±7.8	62.5±9.6	
Sex, n (%)						0.59
Male	246(69.9)	109(69.9)	108(67.1)	8(80.0)	21(84.0)	
Female	106(30.1)	47(30.1)	53(32.9)	2(20.0)	4(16.0)	
Histology, n (%)						0.70
Squamous cell carcinoma	118(33.5)	54(34.6)	52 (32.3)	4(40.0)	8(32.0)	
Adenocarcinoma	181(51.4)	81(51.9)	82(50.9)	3(30.0)	15(60.0)	
Other	53(15.1)	21(13.4)	27(16.8)	3(30.0)	2(8.0)	
Stage, n (%)						-
I	53(15.1)	47(30.1)	(0.0)	0 (0.0)	6(24.0)	
II	77(21.9)	73(46.8)	(0.0)	2(20.0)	2(8.0)	
IIIA	48(13.6)	36(23.1)	8(5.0)	1(10.0)	3(12.0)	
IIIB	54(15.3)	(0.0)	47(29.2)	5(10.0)	2(8.0)	
IV	120(34.1)	(0.0)	106(65.8)	2(10.0)	12(48.0)	
ECOG performance status						0.28
0–1	343(97.4)	154(98.7)	155(96.2)	9(90.0)	24(96.0)	
≥2	9(2.6)	2(1.3)	6(3.7)	1(10.0)	1(4.0)	
Smoking status						0.29†
Never smokers	170(48.3)	80(51.3)	73(45.3)	4(40.0)	13(52.0)	
Ever smokers	182(51.7)	76(48.7)	88(54.7)	6(60.0)	12(48.0)	
<25 pack-years	54(15.3)	27(17.3)	23(14.3)	1(10.0)	3(12.0)	
25–50 pack-years	94(26.7)	37(23.7)	46(28.6)	5(50.0)	6(24.0)	
>50 pack-years	34(9.7)	12(7.7)	19(11.8)	0 (0.0)	3(12.0)	
Radiation therapy						<0.0001
Never received	246(69.9)	124(79.5)	90(55.9)	-	22(88.0)	
Ever received	106(30.1)	32(20.5)	71(44.1)	-	3(12.0)	

Abbreviations: ECOG, Eastern Cooperative Oncology Group; SD, stand deviation.

*P values refer to the comparison between early stage patients with surgery and advanced patients with chemotherapy. Student's t-test was used to compare age as a continuous variable; chi-square test was used to compare categorical variables, except for ECOG performance status, where Fisher's exact test was used.

† Comparisons between never smokers and ever smokers.

### Associations of SNPs with chemotherapy response

Of the 161 advanced NSCLC patients that received evaluable platinum-based chemotherapy, the number of patients with CR, PR, SD or PD was 0, 44, 65 and 52, respectively. The overall clinical benefit (CR+PR+SD) rate was 67.7% (109/161). *BRCA1* rs1799966, *MDR1/ABCB1* rs1045642 and clinical stage were significantly associated with clinical benefit (Table 2). The patients carrying *BRCA1* minor allele C (TC+CC) had significantly more chances of achieving clinical benefit than the patients carrying rs1799966 TT genotype (75.2% *vs.* 55.0%, OR = 0.402, 95%CI = 0.204−0.794, p = 0.008). Similarly, patients carrying *MDR1/ABCB1* rs1045642 minor allele A (GA +AA) had more clinical benefit than patients carrying rs1045642 GG genotype (74.5% *vs.* 58.2%, OR = 0.478, 95%CI = 0.244−0.934, p = 0.030). In addition, the patients with clinical stage IV had significantly less chances of achieving clinical benefit than those with clinical stage III (61.3% *vs.* 80.0%, OR = 2.523, 95%CI = 1.171−5.437, p = 0.016). Furthermore, the associations continued to be significant in multivariate logistic regression analysis, which suggested that *BRCA1* rs1799966 (TC+CC *vs.* TT), *MDR1/ABCB1* rs1045642 (GA+AA *vs.* GG) and clinical stage (IV *vs.* III) were independent factors affecting response to chemotherapy (*BRCA1* rs1799966: adjusted OR = 0.410, 95% CI = 0.203−0.831, adjusted p = 0.013; *MDR1/ABCB1* rs1045642: adjusted OR = 0.488, 95% CI = 0.242−0.984, adjusted p = 0.045; clinical stage: adjusted OR = 2.698, 95% CI = 1.214−5.996, adjusted p = 0.015). No significant associations were observed between the other four SNPs (*ERCC1* rs11615, *XPD/ERCC2* rs13181, *XRCC1* rs25487, *XRCC3* rs1799794) or other characteristics (age, sex, histology, chemotherapy regimens, performance status, smoking status, and radiation therapy) and clinical benefit (p>0.05 for all).

**Table 2:** Associations of genotype and clinical stage with clinical benefit of chemotherapy among advanced NSCLC patients (N = 161).

	CR+PR+SD, n (%)	PD, n (%)	*P*	OR (95% CI)	Adjusted *P* *	Adjusted OR* (95% CI)
*BRCA1* rs1799966						
TT	33 (55.0)	27 (45.0)		1.00		1.00
TC+CC	76 (75.2)	25 (24.8)	**0.008**	**0.402 (0.204**–**0.794)**	**0.013**	**0.410 (0.203**–**0.831)**
*MDR1/ABCB1* rs1045642						
GG	39 (58.2)	28 (41.8)		1.00		1.00
GA +AA	70 (74.5)	24 (25.5)	**0.030**	**0.478 (0.244**–**0.934)**	**0.045**	**0.488 (0.242**–**0.984)**
Clinical stage						
III	44 (80.0)	11 (20.0)		1.00		1.00
IV	65 (61.3)	41 (38.7)	**0.016**	**2.523 (1.171**–**5.437)**	**0.015**	**2.698 (1.214**–**5.996)**
*ERCC1* rs11615						
GG	58 (63.7)	33 (36.3)		1.00		
GA+AA	51 (72.9)	19 (27.1)	0.220	0.655 (0.332–1.290)		
*XPD/ERCC2* rs13181						
TT	87 (68.0)	41 (32.0)		1.00		
TG+GG	22 (66.7)	11 (33.3)	0.887	1.061 (0.470–2.393)		
*XRCC1* rs25487						
CC	70 (68.6)	32 (31.4)		1.00		
CT+TT	39 (66.1)	20 (33.9)	0.741	1.122 (0.567–2.219)		
*XRCC3* rs1799794						
CC	39 (63.9)	22 (36.1)		1.00		
CT+TT	70 (70.0)	30 (30.0)	0.425	0.760 (0.387–1.493)		

Abbreviations: CR, complete response; OR, odds ratio; PD, progressive disease; PR, partial response; SD, stable disease.

*Adjusted for *BRCA1* rs1799966, *MDR1/ABCB1* rs1045642 and clinical stage, adjusted ORs and P values were obtained from multivariate logistic regression analysis by forward stepwise method.

### Associations of SNPs with OS of patients with advanced NSCLC

**Figure 1:**
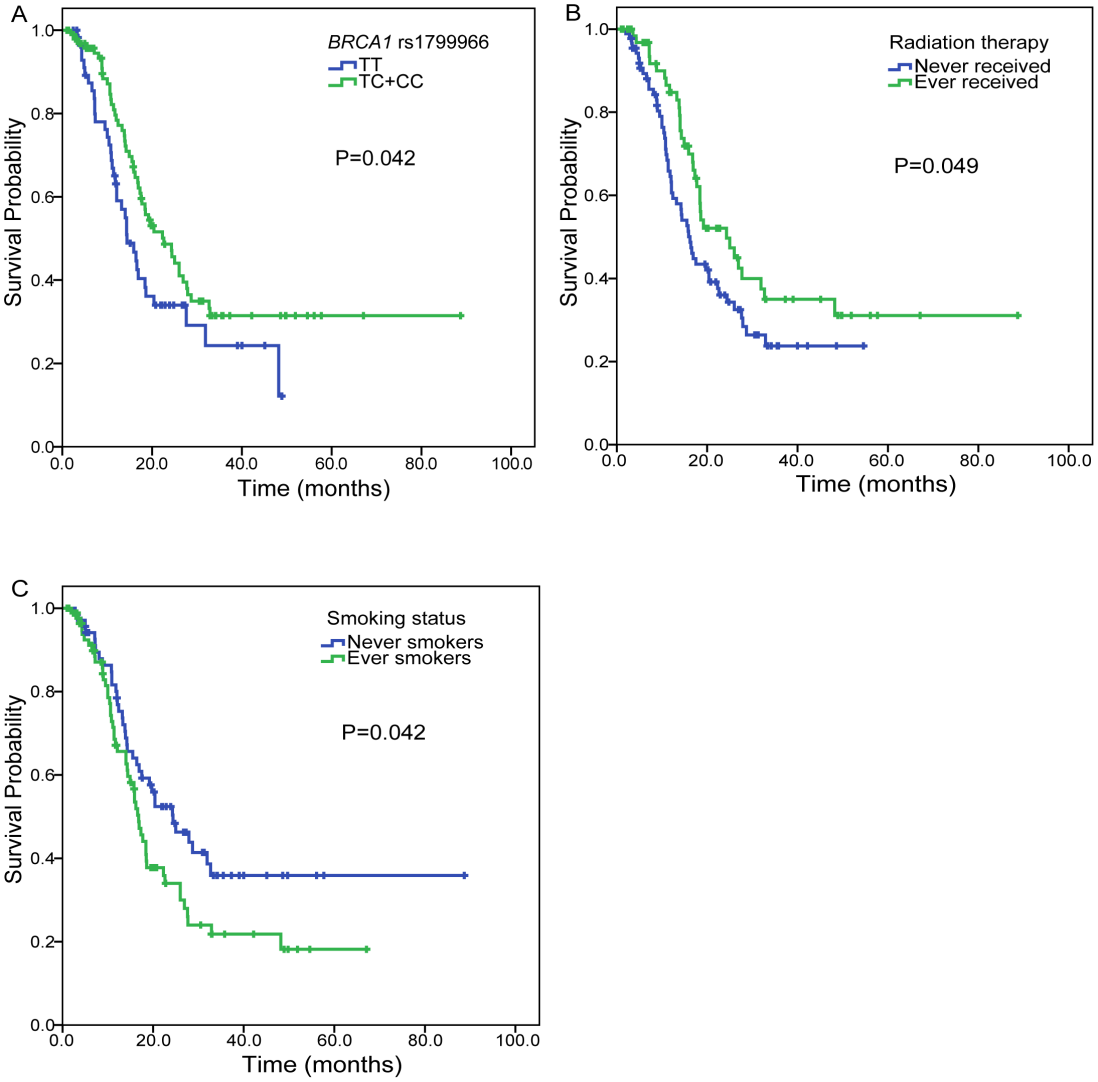
Factors associated with overall survival of advanced NSCLC patients. (A) *BRCA1* rs1799966, (B) radiation therapy, (C) smoking status.

Univariate analyses using Kaplan-Meier curves and log-rank tests indicated that *BRCA1* rs1799966 (TC+CC *vs.* TT genotypes), radiation therapy (ever *vs.* never), and smoking status (never *vs.* ever) were significantly associated with an increased OS of advanced NSCLC patients with log-rank values of p = 0.042, 0.049, and 0.042, respectively (Fig. 1). The stepwise multivariate Cox analysis showed that *BRCA1* rs1799966 (TC+CC *vs.* TT: HR = 0.617, 95% CI = 0.402−0.948, p = 0.028), radiation therapy (ever *vs.* never: HR = 0.611, 95% CI = 0.396−0.944, p = 0.027), and smoking (never *vs.* ever: HR = 0.574, 95% CI = 0.373−0.882, p = 0.011) independently predicted favorable survival of the advanced NSCLC patients. No significant associations were observed between the other SNPs or other characteristics and OS (log-rank p>0.05 for all).

**Figure 2:**
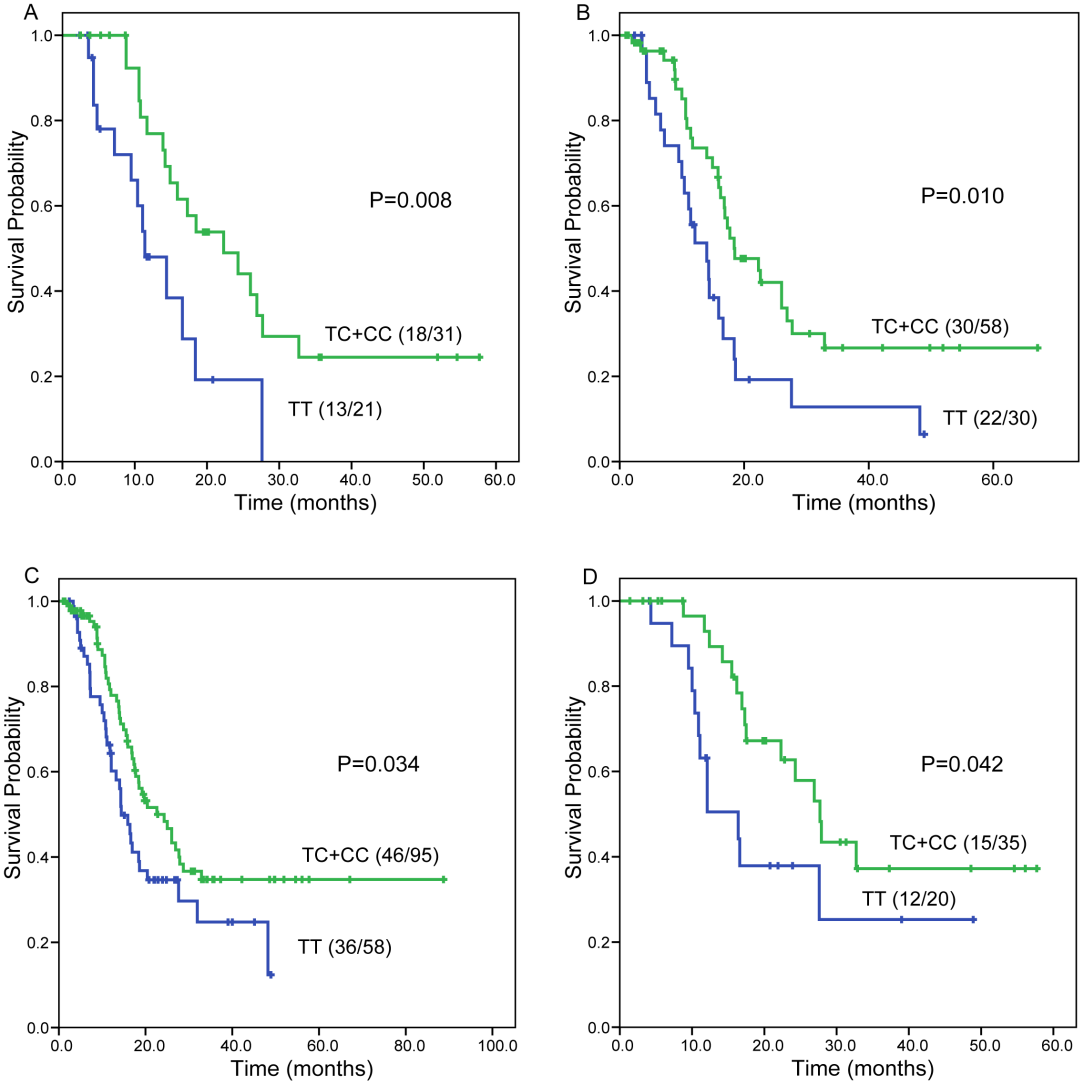
Subgroup analysis results of overall survival in advanced NSCLC patients according to *BRCA1* rs1799966 genotypes (TT *vs.* TC+CC). (A) Squamous cell carcinoma group, (B) ever smokers group, (C) ECOG performance status  = 1 group, (D) stage III group. Number in parenthesis, number of deaths/number of cases.

We further examined the association of *BRCA1* rs1799966 with survival in subgroup analyses by categorizing the patients according to histology, smoking status, ECOG performance status, and stage. In with squamous cell carcinoma, those ever smoked, those with ECOG performance status of 1, or those at stage III, *BRCA1* rs1799966 TC+CC genotypes predicted a longer OS than the TT genotype (log-rank p<0.05 for all) (Fig. 2). After adjusting for age, sex, histology, stage, ECOG performance status, smoking status, and radiation therapy in the Cox model, *BRCA1* rs1799966 TC+CC genotypes were associated with a lower risk of death in patients with squamous cell carcinoma (HR = 0.324, 95% CI = 0.137−0.765, p = 0.010), patients ever smoked (HR = 0.406, 95% CI = 0.221−0.743, p = 0.004), or those with ECOG performance status of 1 (HR = 0.563, 95% CI = 0.353−0.898, p = 0.016).

### Associations of SNPs with postoperative OS of patients with early stage NSCLC

**Figure 3:**
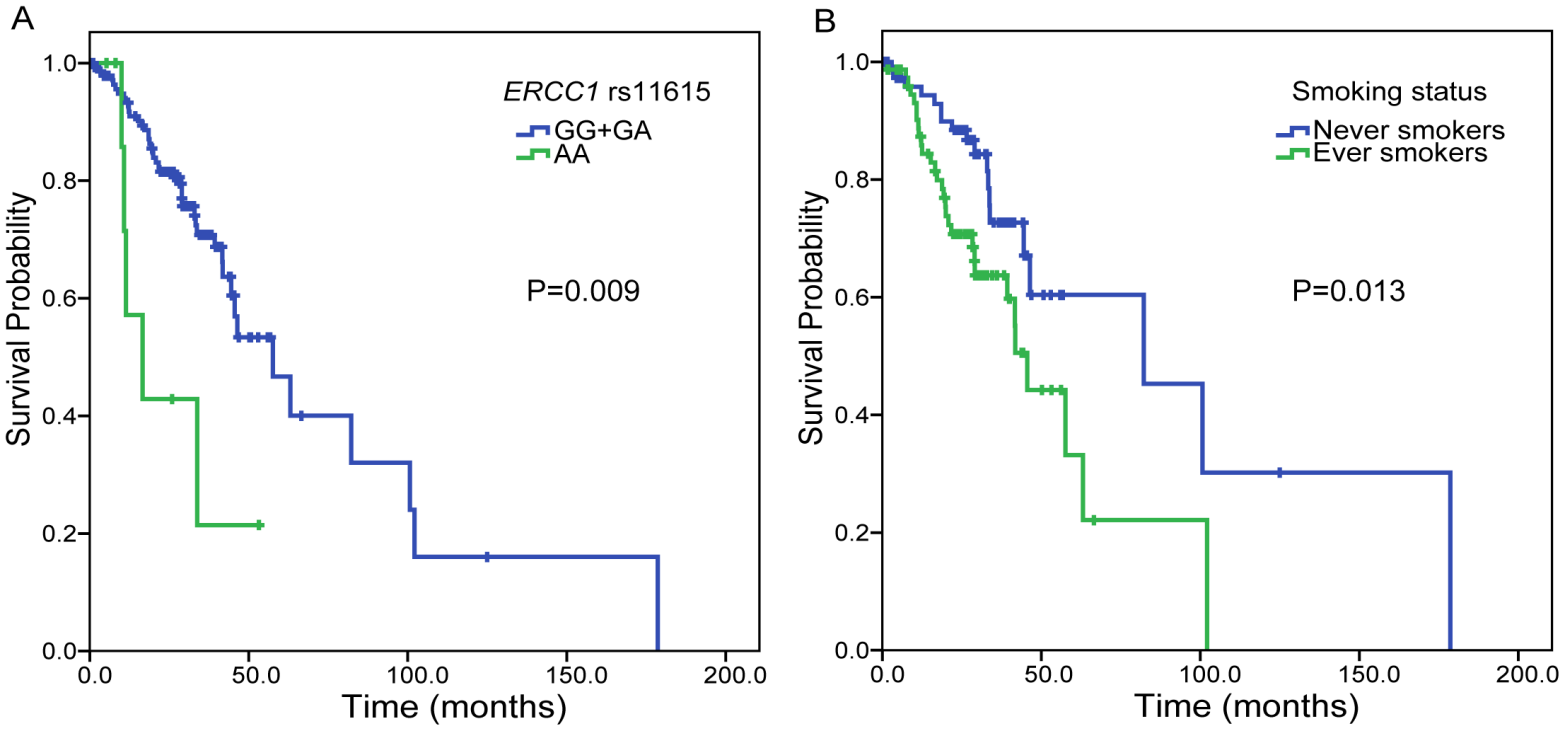
Factors associated with overall survival of early stage NSCLC patients. (A) *ERCC1* rs11615, (B) smoking status.

*ERCC1* rs11615 and smoking status were statistically significantly associated with OS of early stage NSCLC patients (log-rank p = 0.009 and 0.013, respectively) (Fig. 3). In the stepwise multivariate Cox analysis, patients with *ERCC1* rs11615 AA genotype had an increased risk of death (deaths/cases: 5/9 for AA *vs.* 44/147 for GG+GA, HR = 3.087, 95% CI = 1.197−7.961, p = 0.020,). Smoking was also associated with worse survival (deaths/cases: 30/76 for ever smokers *vs.* 19/80 for never smokers, HR = 1.896, 95% CI = 1.040−3.455, p = 0.037). We did not observe any significant associations of the other SNPs or other characteristics with OS (log-rank p>0.05 for all).

**Figure 4:**
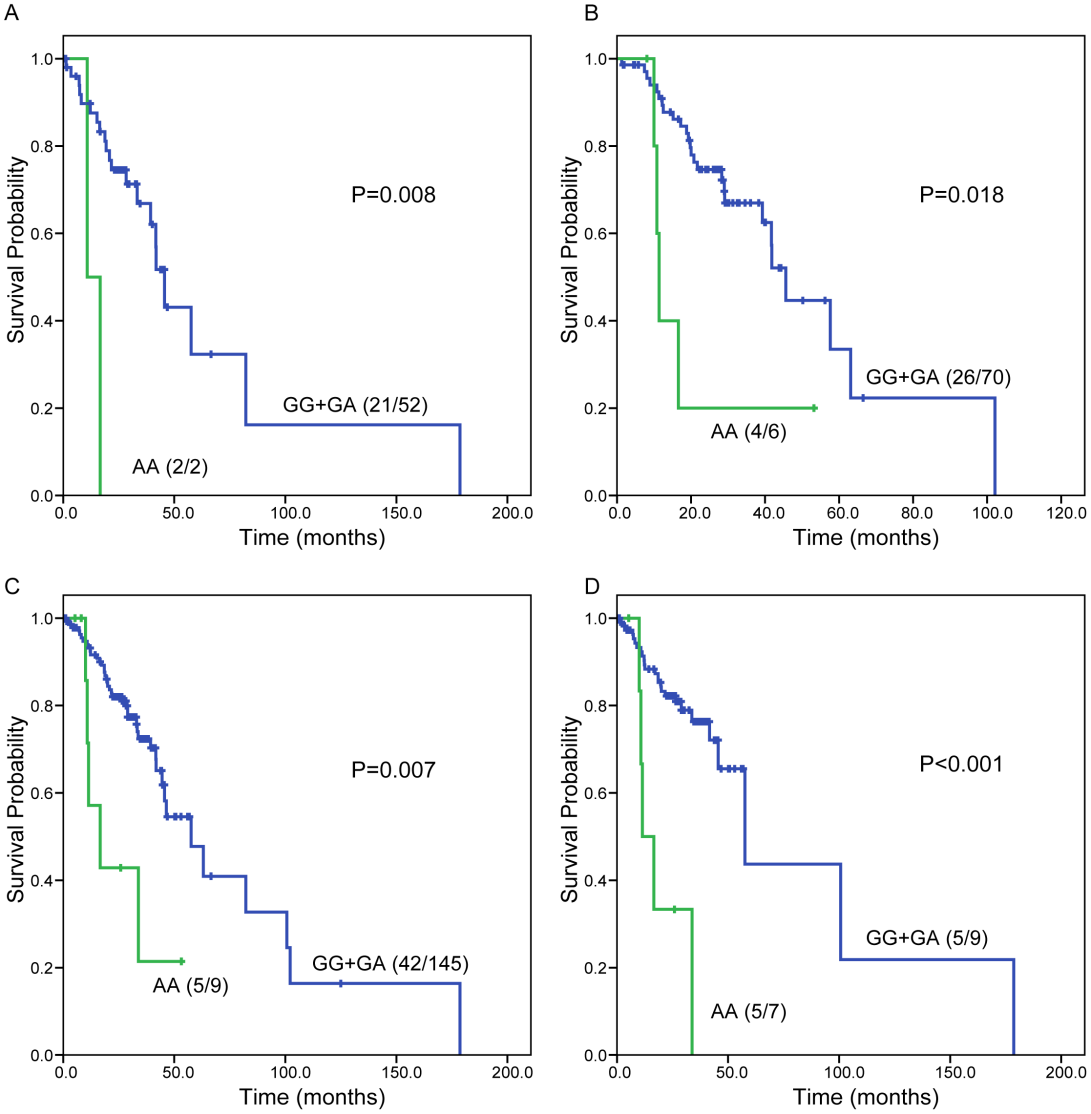
Subgroup analysis results of overall survival in early stage NSCLC patients according to *ERCC1* genotypes (GG+GA vs. AA). (A) Squamous cell carcinoma group, (B) ever smokers group, (C) ECOG performance status  = 1 group, (D) never receiving radiation therapy group. Number in parenthesis, number of deaths/number of cases.

Further stratification analyses in early stage NSCLC patients indicated that *ERCC1* rs11615 AA genotype predicted a significant poorer OS than GG+GA genotypes in patients with squamous cell carcinoma (log-rank p = 0.004), those ever smoked (log-rank p = 0.018), those with ECOG performance status of 1 (log-rank p = 0.007), or those never receiving radiation therapy (log-rank p<0.001) (Fig. 4). After adjusting for age, sex, histology, stage, ECOG performance status, smoking status, radiation therapy, and adjuvant chemotherapy in the Cox model, *ERCC1* rs11615 AA genotype predicted a higher risk of death in early stage patients with squamous cell carcinoma (HR = 6.633, 95% CI = 1.184−37.164, p = 0.031), those ever smoked (HR = 3.324, 95% CI = 1.040−10.627, p = 0.043), those with ECOG performance status of 1 (HR = 2.835, 95% CI = 1.063−7.560, p = 0.037), or those never receiving radiation therapy (HR = 5.381, 95% CI = 1.857−15.593, p = 0.002).

## Discussion

In this study, we evaluated relationships between six SNPs with chemotherapy response and survival of NSCLC in a Han Chinese population. We observed that the minor allele C of *BRCA1* rs1799966 from the DSBR pathway was associated with a better treatment response and a longer survival in advanced NSCLC patients underwent platinum-based chemotherapy. In addition, minor allele A of *MDR1/ABCB1* rs1045642 was significantly associated with clinical benefit of chemotherapy in advanced NSCLC patients. For early NSCLC patients, carriers of *ERCC1* rs11615 AA genotype had an increased risk of death.

*BRCA1* gene belongs to the NER system [23], which is the major repair system that reduces platinum-induced DNA damage. *BRCA1* involves in many activities including homologous recombination, nonhomologous end joining, and mismatch repair [24]. BRCA1 protein is a nuclear phosphoprotein with multiple roles not only in DNA damage repair but also in cell cycle checkpoint or cell death machinery [24], [25]. *In vitro* studies have reported that BRCA1 can regulate chemotherapy agent sensitivity; for example, the absence of BRCA1 causes high sensitivity to cisplatin [24]. BRCA1 expression is a reliable indicator of chemoresistance in NSCLC patients receiving treatment by DNA-damaging agents such as platinum [26]–[28]. Patients with lower BRCA1 expression have better survival in platinum-based neoadjuvant chemotherapy [26]–[28], which is further confirmed by a recent meta-analysis [29]. Genetic polymorphisms may affect the protein expression, structure, and/or function; however, previous studies have rarely investigated the associations between *BRCA1* polymorphisms with NSCLC survival. One study in a Korean population reported no association between *BRCA1* rs1799966 and NSCLC survival; however, they did find *BRCA1* haplotype AACC (rs1799966-rs8176199-rs8067269-rs2070833) predicted shorter survival among advanced NSCLC patients receiving platinum-based chemotherapy [18]. In our analysis, we observed *BRCA1* rs1799966 minor allele C was significantly associated with better chemotherapy response and longer survival in advanced NSCLC patients. rs1799966 is a nonsynonymous SNP in the coding region of the COOH-terminal domain, termed as BRCT, which is essential for homologous recombination in repairing DNA double-stranded breaks [30]. The amino acid change from proline to leucine at position 871 of rs799917 leads to a non-conservative change of the unique structural properties of the BRCA1 protein. In addition, this SNP is in linkage disequilibrium with rs799917, which lies in the middle of a strongly conserved region of the *BRCA1* gene [31].

ERCC1 is another important enzyme in the NER pathway of DNA repair, and is responsible for the 5′ incision of damaged DNA [32]. Previous studies have demonstrated that ERCC1 expression is related to clinical benefit of platinum-based chemotherapy [5], [8], [33], [34], and could be used as a biomarker for NSCLC treatment. The variant genotype in *ERCC1* polymorphisms may destroy or alter the repair functions possibly through changing the expression levels. rs11615 is a synonymous SNP located in codon 118 of the *ERCC1* gene, which encodes the amino acid asparagines. The variant allele of rs11615 may results in altered mRNA level [35]. Studies conducted in different populations showed that the GG genotype of *ERCC1* rs11615 was associated with a better survival in advanced NSCLC patient [36], [37]. In contrary, a study conducted in Japan did not find significant associations between *ERCC1* rs11615 and survival [38]. Furthermore, a meta-analysis including 17 studies could not confirm the association of *ERCC1* rs11615 with survival, neither in the overall nor ethnic stratified analyses [39]. The inconsistent results of previous studies may be due to ethnic differences, the stage of the disease, and/or therapeutic variations. Few studies ever investigated the association between rs11615 and survival of early stage NSCLC. In the present study, we detected that the AA genotype of rs11615 was associated with poorer OS in early stage NSCLC patients. One important meta-analysis concluded that high ERCC1 was associated with significantly worse OS in platinum-treated NSCLC patients [40]. These evidences indicate that the variant allele genotype (AA) of rs11615 is related to high expression of ERCC1. However, the effects of radiation therapy are complex. A fraction of our early stage NSCLC patients (32/156, 20.5%) received radiation therapy, thus we cannot rule out the possible influence of radiation therapy on the association of *ERCC1* rs11615 with survival.

One reason of treatment failure in cancer patients is drug resistance, which can be influenced by factors affecting the efflux and influx of chemotherapeutics across the cell membrane. MDR1/ABCB1 is responsible for the efflux of many chemotherapeutics through the cell membrane by hydrolysis of ATP [41]. rs1045642 has a G>A change at cDNA position 3435 in exon 26 and plays a role in the P-gp function [20]. However, results from studies of *MDR1/ABCB1* rs1045642 and platinum-based chemotherapy response of NSCLC are inconsistent in different populations [36], [42]. Recent meta-analyses confirmed this SNP was associated with chemotherapy response in overall and Asian populations [39], [43]. However, we obtained opposite results from the meta-analysis findings, showing *MDR1/ABCB1* variant allele carriers had more clinical benefits of chemotherapy. The discrepancy may possibly be explained by potential confounders such as radiation therapy.

Not surprisingly, smoking was associated with NSCLC prognosis in both early and advanced stage patients. Smoking causes impaired lung function, and is the well established risk factor of NSCLC occurrence and poor prognosis. Smoking can cause DNA damages such as DNA adducts and double-strand DNA break lesions [44]. It can also interfere with DNA repair function, thus greatly affecting patient outcomes.

The current study has several limitations. First are the relatively small sample size and a short follow-up period, especially in subgroup analyses, therefore raising the issue of false positive findings. Our results did not reach statistical significance after correcting for multiple comparisons, and these findings need to be further validated. Second, we collected OS data instead of disease-specific survival since it is difficult to confirm the real cause of death in some patients. Third, we only selected limited number of SNPs based on their functions, while other polymorphisms in these genes may affect NSCLC chemotherapy response and prognosis. Last, NSCLC is a heterogeneous group of neoplasm, and multiple mechanisms may determine the response and prognosis, we were unable to account all factors in the current analysis.

To conclude, our study provided further evidences that polymorphisms in DNA repair pathway genes and *MDR1* gene could influence treatment response and survival of NSCLC patients. The mechanisms of how the differences in SNP nucleotide sequences or tandem repeats can affect gene transcription remain largely unclear. Further large-scale studies are needed to elucidate the functions and mechanisms that may help in improving the outcomes of NSCLC patients.

## Supporting Information

Table S1 Polymorphism information, primers and probes for genotyping.
(DOC)

Table S2 Genotype distributions of SNPs in NSCLC patients.
(DOC)

Dataset S1 (XLS)
