# Supplementary material for: Associations of Polymorphisms in DNA Repair Genes and MDR1 Gene with Chemotherapy Response and Survival of Non-Small Cell Lung Cancer
Source: PLoS One. 2014 Jun 16;9(6):e99843. doi: 10.1371/journal.pone.0099843 (PMC4059653; doi:10.1371/journal.pone.0099843)
Supplement: Table S1 — Polymorphism information, primers and probes for genotyping. (DOC) [file pone.0099843.s001.doc]

Table S1. Polymorphism information, primers and probes for genotyping.

| **SNP** | **Gene** | **Chr** | **Chr position*** | **Base (amino acid) change** | **HapMap MAF** | **Primer (5'🡪3')** | **Probe (5'🡪3')** |
| --- | --- | --- | --- | --- | --- | --- | --- |
| rs11615 | *ERCC1* | 19q13.32 | 45923653 | G>A | 0.243 | GTCATCCCTATTGATGGCTTCTG | FAM-TCGTGCGCAACGTGCCCT-TAMRA |
|  |  |  |  | (Asn118Asn) |  | GGGAATTACGTCGCCAAATTC | HEX-TCGTGCGCAATGTGCCCTG-TAMRA |
| rs13181 | *XPD/*  *ERCC2* | 19q13.3 | 45854919 | T>G | 0.095 | AGTCACCAGGAACCGTTTATGG | FAM-CTATCCTCTTCAGCGTC-MGB |
|  |  |  |  | (Lys751Gln) |  | TCTGTTCTCTGCAGGAGGATCA | HEX-TCCTCTGCAGCGTC-MGB |
| rs25487 | *XRCC1* | 19q13.2 | 44055726 | C>T | 0.252 | AAGGAGTGGGTGCTGGACTGT | FAM-CTGCCCTCCCAGAGGTAAGGCCT -TAMRA |
|  |  |  |  | (Arg399Gln) |  | TCTGACTCCCCTCCAGATTCC | TET- CTGCCCTCCCGGAGGTAAGGC -TAMRA |
| rs1799794 | *XRCC3* | 14q32.3 | 104179267 | C>T | 0.478 | GTCCACTGACGGATAACAGACTCA | FAM- CCTCTGTGCACACCCTGCTGAGA-TAMRA |
|  |  |  |  | (5’-UTR) |  | CCTAATCAGCTGTCAAGGGTGAT | HEX-CCTCTGTGCACATCCTGCTGAGAAC-TAMRA |
| rs1799966 | *BRCA1* | 17q21 | 41223094 | T>C | 0.327 | ATACCATCTTCAACCTCTGCATTG | FAM-TCTGCCCAGAGTCCAGCTGCTG-TAMRA |
|  |  |  |  | (Ser1613Gly) |  | CCCTGCTCACACTTTCTTCCA | HEX-CTGCCCAGGGTCCAGCTGCT-TAMRA |
| rs1045642 | *MDR1/ABCB1* | 7q21.12 | 78398146 | G>A | 0.374 | AGTGACTCGATGAAGGCATGTATG | FAM-TTGCTGCCCTCACGATCTCTTCCT-TAMRA |
|  |  |  |  | (Ile1145Ile) |  | TGACTGCAGCATTGCTGAGAA | HEX-TTGCTGCCCTCACAATCTCTTCCTG-TAMRA |

Abbreviations: MAF, minor allele frequency; SNP, single nucleotide polymorphism

Database of single nucleotide polymorphisms (dbSNP), Bethesda (MD): National Center for Biotechnology Information, National Library of Medicine. (dbSNP Build ID: {build ID}. Available from: <http://www.ncbi.nlm.nih.gov/snp/>

*dbSNP Chromosome Report, GRCh37.p10
