# Supplementary material for: Associations of Polymorphisms in DNA Repair Genes and MDR1 Gene with Chemotherapy Response and Survival of Non-Small Cell Lung Cancer
Source: PLoS One. 2014 Jun 16;9(6):e99843. doi: 10.1371/journal.pone.0099843 (PMC4059653; doi:10.1371/journal.pone.0099843)
Supplement: Table S2 — Genotype distributions of SNPs in NSCLC patients. (DOC) [file pone.0099843.s002.doc]

Table S2. Genotype distributions of SNPs in NSCLC patients.

|  |  |  | **N (%)** | | | | |
| --- | --- | --- | --- | --- | --- | --- | --- |
| **Gene** | **rs Number** | **Genotype** | **Total NSCLC**  **(n=352)** | **Surgery, early**  **(n=156)** | **Chemotherapy, advanced**  **(n=161)** | **Refused treatment**  **(n=10)** | **Lost to follow-up**  **(n=25)** |
| *ERCC1* | rs11615 | GG | 202 (57.4) | 86 (55.1) | 91 (56.5) | 10 (100.0) | 15 (60.0) |
|  |  | GA | 126 (35.8) | 61 (39.1) | 55 (34.2) | 0 (0) | 10 (40.0) |
|  |  | AA | 24 (6.8) | 9 (5.8) | 15 (9.3) | 0 (0) | 0 (0) |
| *XPD/ERCC2* | rs13181 | TT | 288 (81.8) | 133 (85.3) | 128 (79.5) | 6 (60.0) | 21 (84.0) |
|  |  | TG | 62 (17.6) | 23 (14.7) | 32 (19.9) | 3 (30.0) | 4 (16.0) |
|  |  | GG | 2 (0.6) | 0 (0) | 1 (0.6) | 1 (10.0) | 0 (0) |
| *XRCC1* | rs25487 | CC | 190 (54.0) | 69 (44.2) | 102 (63.4) | 6 (60.0) | 13 (52.0) |
|  |  | CT | 143 (40.6) | 77 (49.4) | 51 (31.7) | 4 (40.0) | 11 (44.0) |
|  |  | TT | 19 (5.4) | 10 (6.4) | 8 (5.0) | 0 (0) | 1 (4.0) |
| *XRCC3* | rs1799794 | CC | 110 (31.2) | 43 (27.6) | 61 (37.9) | 2 (20.0) | 4 (16.0) |
|  |  | CT | 164 (46.6) | 70 (44.9) | 74 (46.0) | 5 (50.0) | 15 (60.0) |
|  |  | TT | 78 (22.2) | 43 (27.6) | 26 (16.1) | 3 (30.0) | 6 (24.0) |
| *BRCA1* | rs1799966 | TT | 137 (38.9) | 67 (42.9) | 60 (37.3) | 4 (40.0) | 6 (24.0) |
|  |  | TC | 181 (51.4) | 77 (49.4) | 82 (50.9) | 5 (50.0) | 17 (68.0) |
|  |  | CC | 34 (9.7) | 12 (7.7) | 19 (11.8) | 1 (10.0) | 2 (8.0) |
| *MDR1/ABCB1* | rs1045642 | GG | 142 (40.3) | 65 (41.7) | 67 (41.6) | 1 (10.0) | 9 (36.0) |
|  |  | GA | 163 (46.3) | 72 (46.2) | 73 (45.3) | 7 (70.0) | 11 (44.0) |
|  |  | AA | 47 (13.4) | 19 (12.2) | 21 (13.0) | 2 (20.0) | 5 (20.0) |

Abbreviations: NSCLC, non-small-cell lung cancer
